# Supplementary material for: Immunogenicity after a Third COVID-19 mRNA Booster in Solid Cancer Patients Who Previously Received the Primary Heterologous CoronaVac/ChAdOx1 Vaccine
Source: Vaccines (Basel). 2022 Sep 26;10(10):1613. doi: 10.3390/vaccines10101613 (PMC9608216; doi:10.3390/vaccines10101613)
Supplement: Supplementary file 1 [file vaccines-10-01613-s001.zip › vaccines-1889374-supplementary.pdf]

Table of Contents

Contents	Page
**Supplemental tables**	
Table S1: Clinical characteristics of patients with inadequate antibody response defined by anti-RBD IgG less than 300 BAU/ml	2
Table S2: Post-third dose levels of SAR-CoV2 binding antibody stratified by clinical factors	3
Table S3: Vaccine-related reactogenicity compared between CoronaVac/ChAdOx1/mRNA and ChAdOx1/ChAdOx1/mRNA vaccine scheme	4
Table S4: Demographics and clinical characteristics of samples performed omicron neutralization	5

Table S1. Clinical characteristics of patients with inadequate antibody response defined by anti-RBD IgG less than 300 BAU/ml.

No	Age	Sex	Cancer Types	Treatment	Co-morbidity	Anti-RBD IgG (BAU/ml) pre-third dose	Anti-RBD IgG (BAU/ml) post-third dose	Steroid use
1	53	Female	Breast T2N1M0	Adjuvant doxorubicin/cyclophosphamide 4 cycles followed by weekly paclitaxel and trastuzumab	No	48.14	42.80	Pre-medication
2	72	Male	Locally advanced nasopharyngeal cancer	Induction cisplatin/5-FU for 3 cycles then concurrent chemoradiation with cisplatin/5-FU for 3 cycles	No	12.13	54.83	Pre-medication
3	63	Female	Recurrent colon cancer	Palliative oxaliplatin/5-FU	DM, HT, Congenital adrenal hyperplasia	4.25	103.22	Therapeutic steroid
4	65	Female	Recurrent colon cancer	Palliative second-line irinotecan/5-FU	DM, HT	55.04	228.29	Pre-medication

Table S2. Post-third dose levels of SAR-CoV2 binding antibody stratified by clinical factors.

**Clinical factors**	**Cancer patient CoronaVac-ChAdOx1**	**GMT (95%CI) at post-third dose**	***p*-value**
**Overall**	**(n= 44)**	2329.63 (1487.4-3648.76)	
**Age, years, median (IQR)**	57 (48.5-65)		
<60 years	24 (55%)	3267.02 (1888.09-5653.04)	0.131
≥60 years	20 (45%)	1552.56 (733.16-3287.75)	
**Sex**			
Female	24 (55%)	2476.88 (1293.16-4744.12)	0.588
Male	20 (45%)	2164.44 (1107.28-4230.93)	
**Cancer types**			
Breast	18 (41%)	2925.42 (1426.37-5999.91)	0.646
Colorectal	11 (25%)	1940.81 (758.45-4966.34)	
Head Neck	6 (14%)	762.08 (132.26-4391.02)	
Hepato-Biliary-Pancreatic	4 (9%)	2114.43 (230.73-19377.07)	
Esophagus/Gastric/Genitourinary	5 (11%)	6334.79 (1569.76-25564.12)	
**Cancer treatment**			
Chemotherapy	29 (66%)	1558.22 (851.37-2851.94)	**0.013**
Hormonal therapy/Biologics	15 (34%)	5069.41 (3236.82-7939.57)	
**Corticosteroid**			
no/pre-medication	41 (93%)	2460.75 (1570.16-3856.47)	0.625
therapeutic purpose	3 (7%)	1102.28 (3.43-354531.6)	
**Disease status**			
Early	20 (45%)	3055.02 (1629.83-5726.45)	0.664
Locally advanced	8 (18%)	1809.22 (448.38-7300.28)	
De novo metastasis	10 (23%)	2810.11 (1146.55-6887.38)	
Recurrence	6 (14%)	967.24 (126.31-7406.61)	
**Co-morbidity**			
Diabetes	6 (14%)	1487.06 (226.3-9771.71)	0.473
Hypertension	12 (27%)	1762.26 (661.69-4693.39)	0.343
**Type of third vaccine**			
BNT162b2 (Pfizer)	20 (45%)	2037.04 (1028.7-4033.76)	0.540
mRNA-1273 (Moderna)	24 (55%)	2605.31 (1376.54-4930.96)	

Table S3. Vaccine-related reactogenicity compared between CoronaVac/ChAdOx1/mRNA and ChAdOx1/ChAdOx1/mRNA vaccine scheme.

	**CoronaVac/ChAdOx1/mRNA (n=42)**								**ChAdOx1/ChAdOx1/mRNA (n=86)**								***p*-value***
	**n**	**(%)**	**Mild**	**(%)**	**Moderate**	**(%)**	**Severe**	**(%)**	**n**	**(%)**	**Mild**	**(%)**	**Moderate**	**(%)**	**Severe**	**(%)**	
**Any reactions**	20	(48)							56	(65)							0.058
**Local reactions**																	
Pain	20	(48)	9	(21)	11	(26)	0	(0)	54	(63)	26	(30)	28	(33)	0	(0)	0.103
Tenderness	15	(36)	8	(19)	7	(17)	0	(0)	50	(58)	30	(35)	20	(23)	0	(0)	**0.017**
Induration	5	(12)	5	(12)	0	(0)	0	(0)	19	(22)	16	(19)	3	(3)	0	(0)	0.166
Erythema	1	(2)	0	(0)	1	(2)	0	(0)	2	(2)	2	(2)	0	(0)	0	(0)	0.984
**Systemic reactions**																	
Fever	2	(5)	1	(2)	1	(2)	0	(0)	11	(13)	8	(9)	2	(2)	1	(1)	0.219
Headache	7	(17)	2	(5)	5	(12)	0	(0)	27	(31)	14	(16)	13	(15)	0	(0)	0.076
Myalgia	11	(26)	7	(17)	4	(10)	0	(0)	37	(43)	27	(31)	9	(10)	1	(1)	0.065
Fatigue	8	(19)	6	(14)	2	(5)	0	(0)	33	(38)	31	(36)	2	(2)	0	(0)	**0.028**
Nausea/vomitting	0	(0)	0	(0)	0	(0)	0	(0)	5	(6)	5	(6)	0	(0)	0	(0)	0.171
Diarrhea	3	(7)	3	(7)	0	(0)	0	(0)	5	(6)	5	(6)	0	(0)	0	(0)	0.717
Arthralgia	5	(12)	5	(12)	0	(0)	0	(0)	8	(9)	6	(7)	1	(1)	1	(1)	0.757
Lymphadenopathy	5	(12)	5	(12)	0	(0)	0		1	(1)	1	(1)	0	(0)	0	(0)	**0.014**

* comparison of adverse reaction events between two vaccination regimens

Table S4. Demographics and clinical characteristics of samples performed omicron neutralization.

Primary series of vaccination	Cancer patient CoronaVac-ChAdOx1 (n= 44)		Cancer patient ChAdOx1-ChAdOx1 (n= 40)	
**Age, years, median (IQR)**	57	(48.5-65)	56.5	(50-64)
**Sex**				
Female	24	(55%)	23	(58%)
Male	20	(45%)	17	(43%)
**BMI, kg/m2, median (IQR)**	21.7	(19.5-25.5)	22.4	(19.8-25.6)
**Cancer types**				
Breast	18	(41%)	21	(53%)
Colorectal	11	(25%)	12	(30%)
Head Neck	6	(14%)	0	(0%)
Hepato-Biliary-Pancreatic	4	(9%)	2	(5%)
Esophagus/Gastric	3	(7%)	1	(3%)
Genitourinary	2	(5%)	1	(3%)
Lung	0	(0%)	3	(8%)
Other	0	(0%)	0	(0%)
**Cancer treatment before third dose**				
Chemotherapy	29	(66%)	23	(58%)
Hormonal therapy/Biologics	15	(34%)	17	(43%)
**Corticosteroid**				
No/pre-medication	41	(93%)	40	(100%)
Therapeutic purpose	3	(7%)	0	(0%)
**Disease status**				
Early	20	(45%)	20	(50%)
Locally advanced	8	(18%)	5	(13%)
De novo metastasis	10	(23%)	12	(30%)
Recurrence	6	(14%)	3	(8%)
**Co-morbidity**				
Diabetes	6	(14%)	7	(18%)
Hypertension	12	(27%)	8	(20%)
Cardiovascular disease	3	(7%)	1	(3%)
Respiratory tract disease	1	(2%)	2	(5%)
**Interval between first to second vaccine, days**	24	(21-28)	70	(56-84)
**Interval between second to third vaccine, days**	127.5	(113.5-137)	115.5	(103.5-135)
**Interval between third dose to blood collection, days**	14	(14-14)	14	(14-14)
**Type of third vaccine**				
BNT162b2 (Pfizer)	20	(45%)	17	(43%)
mRNA-1273 (Moderna)	24	(55%)	23	(58%)
